# Supplementary material for: Antiferromagnetism-driven two-dimensional topological nodal-point superconductivity
Source: Nat Commun. 2023 Feb 4;14:614. doi: 10.1038/s41467-023-36201-z (PMC9899283; doi:10.1038/s41467-023-36201-z)
Supplement: Supplementary file 1 — SUPPLEMENTAL MATERIAL: Antiferromagnetism-driven two-dimensional topological nodal-point superconductivity [file 41467_2023_36201_MOESM1_ESM.pdf]

SUPPLEMENTARY INFORMATION

Antiferromagnetism-driven two-dimensional topological nodal-point superconductivity

Maciej Bazarnik[1,2,*,§], Roberto Lo Conte[1,*,§], Eric Mascot[1,3,*,§], Kirsten von Bergmann[1], Dirk K. Morr[4], Roland Wiesendanger[1]

[1]*Department of Physics, University of Hamburg, D-20355 Hamburg, Germany*
[2]*Institute of Physics, Poznan University of Technology, Piotrowo 3, 60-965 Poznan, Poland*
[3]*School of Physics, University of Melbourne, Parkville, VIC 3010, Australia*
[4]*Department of Physics, University of Illinois at Chicago, Chicago, IL 60607*

***** These authors contributed equally.

§ Corresponding Authors

mbazarni@physnet.uni-hamburg.de; rolocont@physnet.uni-hamburg.de; eric.mascot@unimelb.edu.au

Content:

Supplementary Note 1. The calculated band structure and density of states

Supplementary Note 2. Topology

Supplementary Note 3. Phase diagram

Supplementary Note 4. Modulation of $\alpha^{\pm}$ states along the FM edge

Supplementary Note 1. The calculated band structure and density of states

The calculated band structure and density of states are shown in Supplementary Figure 1. The band structure shows van-Hove singularities between neighboring NPs at $\left(\frac{\pi}{a}, 0\right)$, $\left(0, \frac{\pi}{b}\right)$, and between non-neighboring NPs near $\left(\frac{0.63\pi}{a}, \frac{0.57\pi}{b}\right)$. In Supplementary Figure 2, we present the projections of the bulk band structure onto the momentum axes parallel to each edge type. We also show the band structure for a cylinder geometry, where the system is finite in the direction perpendicular to the edge and periodic parallel to the edge. The cylinder geometry shows a similar band structure to the projected bulk band structure with the addition of edge bands.

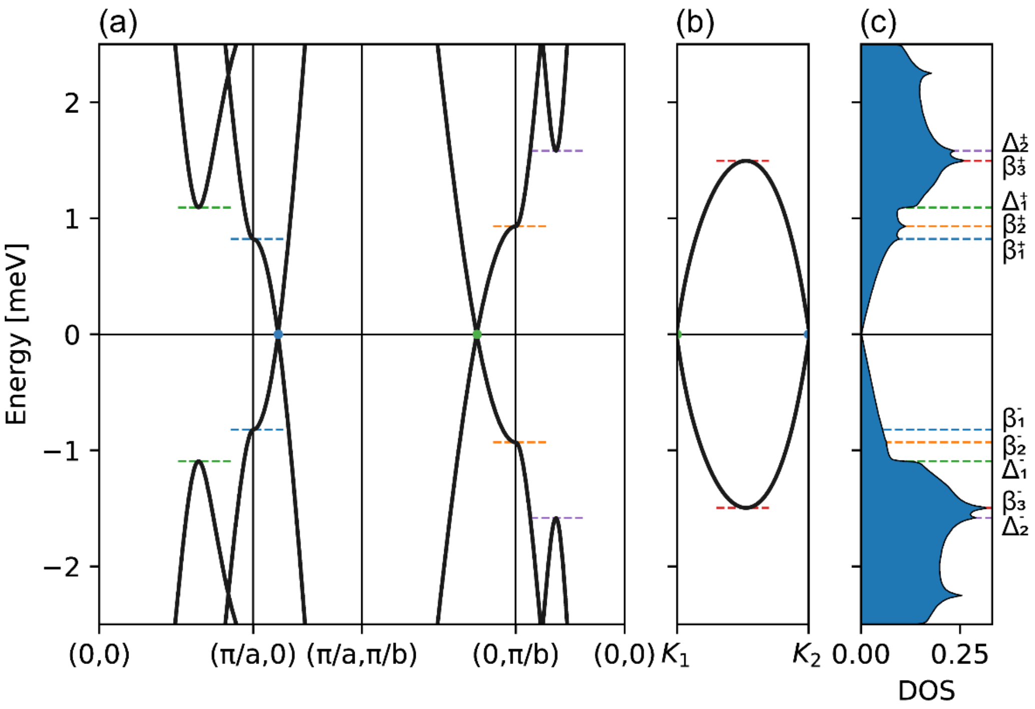

Supplementary Figure 1. Band structure and density of states. (a) Band structure along a high-symmetry path. The blue and orange dashed lines indicate van-Hove singularities and the green and purple dashed lines indicate the coherence peaks. (b) Band structure between two non-neighboring NPs. Dashed red line indicates a van-Hove singularity. (c) Density of states with corresponding van-Hove singularities and coherence peaks indicated by dashed lines.

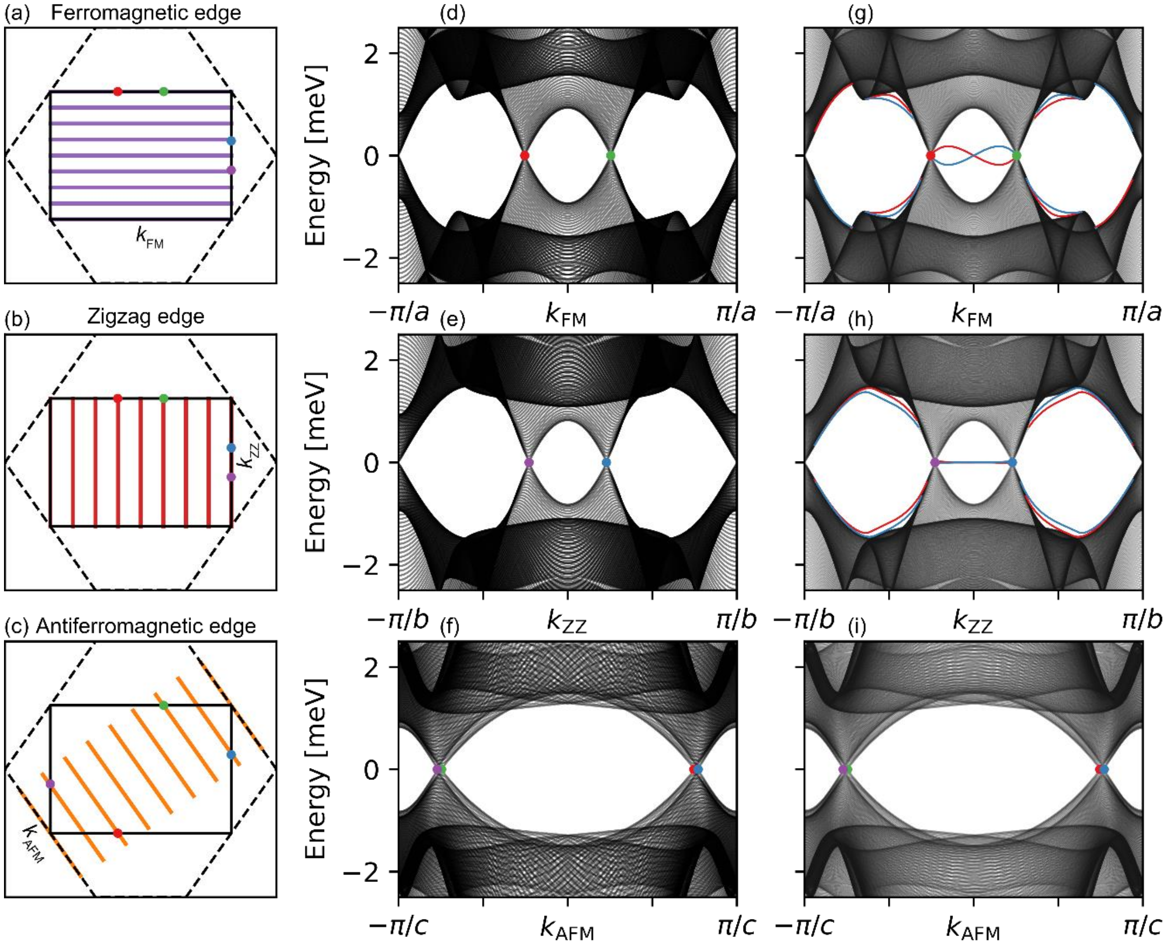

Supplementary Figure 2. Band structure projections. (a-c) Momentum axes parallel to (a) FM, (b) ZZ, and (c) AFM edges. The colored lines indicate the momenta in (d-e). The colored dots indicate the locations of the NPs. The black rectangle shows the magnetic BZ and the dashed rectangle shows the structural BZ. (d-f) Bulk band structure projections for the corresponding edges. (g-i) Band structure for a cylinder geometry for the corresponding edges.

Supplementary Note 2. Topology

The Hamiltonian in Eq. (7) has the symmetries, $T = \eta_x \tau_0 \sigma_y K$, $C = \eta_0 \tau_y \sigma_y K$, and $S = \eta_x \tau_y \sigma_0$. The symmetries square to $T^2 = -1$ and $C^2 = S^2 = 1$, yielding symmetry class DIII [1]. We then transform the Hamiltonian in Eq. (7) to the basis where S is diagonal to get an off-diagonal matrix,

$$U^\dagger S U = \begin{pmatrix} 1 & \\ & -1 \end{pmatrix}, U^\dagger H_{\mathbf{k}} U = \begin{pmatrix} & h_{\mathbf{k}} \\ h_{\mathbf{k}}^\dagger & \end{pmatrix}. \tag{S1}$$

Next, we project the energies to ± 1,

$$Q_{\mathbf{k}} = \sum_n |n_{\mathbf{k}}\rangle \mathrm{sign}(E_{n,\mathbf{k}}) \langle n_{\mathbf{k}}| = \begin{pmatrix} & q_{\mathbf{k}} \\ q_{\mathbf{k}}^\dagger & \end{pmatrix}, \tag{S2}$$

where $E_{n,\mathbf{k}}$ are the energies and $|n_{\mathbf{k}}\rangle$ are the eigenvectors of the Hamiltonian in Eq. (S1). In Supplementary Figure 3, we show the characteristic angle $e^{i\theta_{\mathbf{k}}} = \det(q_{\mathbf{k}})$. The winding of $\theta_{\mathbf{k}}$ around each nodal point defines its topological charge, which is expressed as

$$\nu = \frac{1}{2\pi i} \oint d\mathbf{k} \cdot \mathrm{Tr}\left[q_{\mathbf{k}}^{-1} \nabla_{\mathbf{k}} q_{\mathbf{k}}\right] = \frac{\Delta\theta_{\mathbf{k}}}{2\pi}. \tag{S3}$$

The winding number of the NPs alternate between +1 and -1 along the magnetic BZ boundary and NPs at **k** and -**k** have the same winding number.

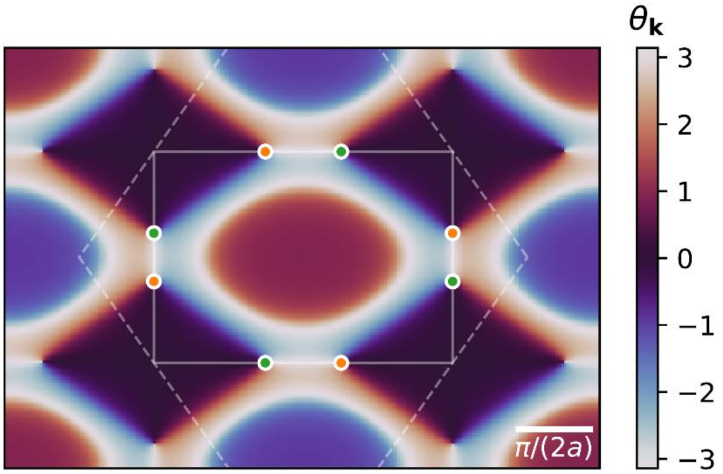

Supplementary Figure 3. Characteristic angle. The magnetic BZ is indicated by solid lines and the structural BZ is indicated by dashed lines. NPs with winding number +1 are marked by green points and NPs with winding number -1 are marked by orange points.

Supplementary Note 3. Phase diagram

To determine to what extent topological nodal points are a general feature of the system, we calculate an energy gap diagram for varying chemical potential, μ, and exchange coupling, *JS*, shown in Supplementary Figure 4 (a). We find that for a wide range of parameters, the system is gapless, which is a necessary requirement for the presence of nodal points, though other types of gap closing could in general also occur. To determine whether the gapless region possesses topological edge states, we start by computing the zero energy LDOS along different edges as shown in Supplementary Figure 4 (b-d). We note this plot reveals a small but non-zero LDOS even in those cases when there is no low-energy edge mode, due to the finite energy width of our calculations and the fact that the bulk system (in the presence of nodal points) possesses an LDOS that increases linearly with energy. The presence of an edge state is reflected by an increase in the spectral weight which depends on the distance between nodal points in momentum space and on the flatness of the edge band. For the parameters used in the main text, indicated by a white circle, the ZZ edge band is the flattest, showing a large spectral weight in Supplementary Figure 4 (c), and the FM edge is dispersive, showing some spectral weight in Supplementary Figure 4 (b). An edge state is not present on the AFM edge, as discussed in the main text, however there is some spectral weight in Supplementary Figure 4 (d) due to the presence of bulk states. Supplementary Figure 4 shows that an edge state is present for a majority of the gapless region. However, which of the three edges exhibits an edge state, as well as the associated spectral weight, varies with μ and *JS*, as the location of the nodal points along the magnetic Brillouin zone is varied.

To better differentiate between bulk states and dispersive or non-dispersive edge states, we present in Supplementary Figure 5 the energy-dependent LDOS as a function of μ for fixed *JS* (these cuts through parameter space correspond to the horizontal white lines in Supplementary Figure 4). For the FM edge in Supplementary Figure 5 (a-c), a dispersive edge band is seen in the gapless parameter range, and the width of this edge band is larger towards the center of the gapless region. Similarly, the ZZ edge in Supplementary Figure 5 (d-f) shows an edge band throughout the gapless region, with a smaller band width. The AFM edge in Supplementary Figure 5 (g-i) shows an edge state only for narrow ranges of the chemical potential. To further identify edge effects, and separate contributions to the LDOS from edge states and bulk states, we subtract the bulk LDOS from the edge LDOS and present the resulting difference in Supplementary Figure 6. This plot further emphasizes the ubiquity of edge states in the gapless region, as expected from the presence of nodal points.

We thus conclude that the presence of topological nodal points, and associated edge states can be found over a wide range of parameters, stressing the generality of the results presented in the main text.

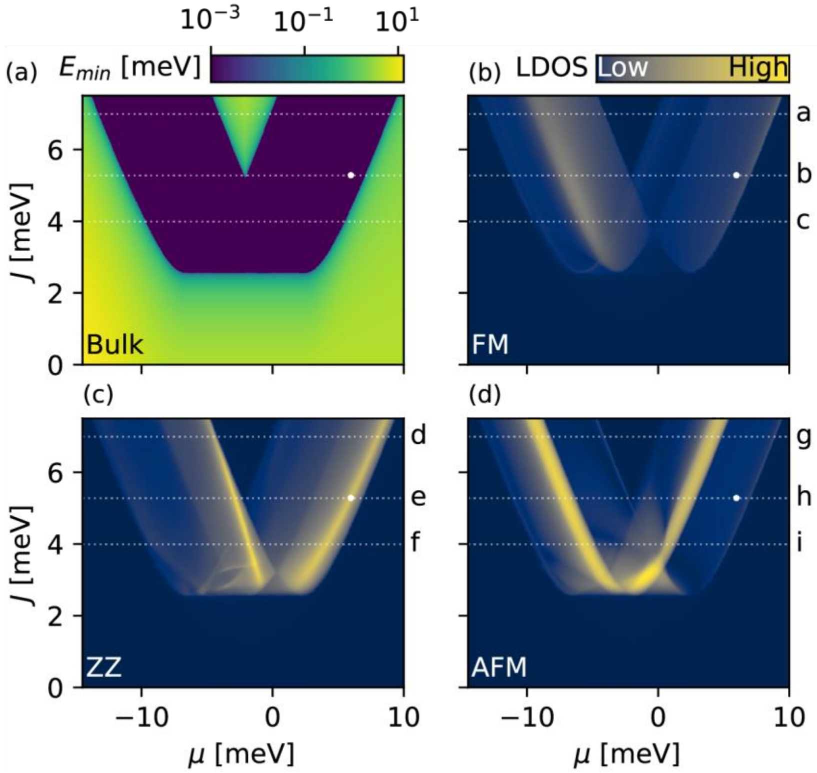

Supplementary Figure 4. Gap diagram and zero-energy LDOS. (a) Lowest energy state of a system with periodic boundary conditions for different parameters. The white circle indicates the parameters used in the main text. The dotted lines indicate the parameters in Supplementary Figure 5 and 6. (b-d) Zero-energy LDOS at (b) a FM edge, (c) a ZZ edge, and (d) an AFM edge.

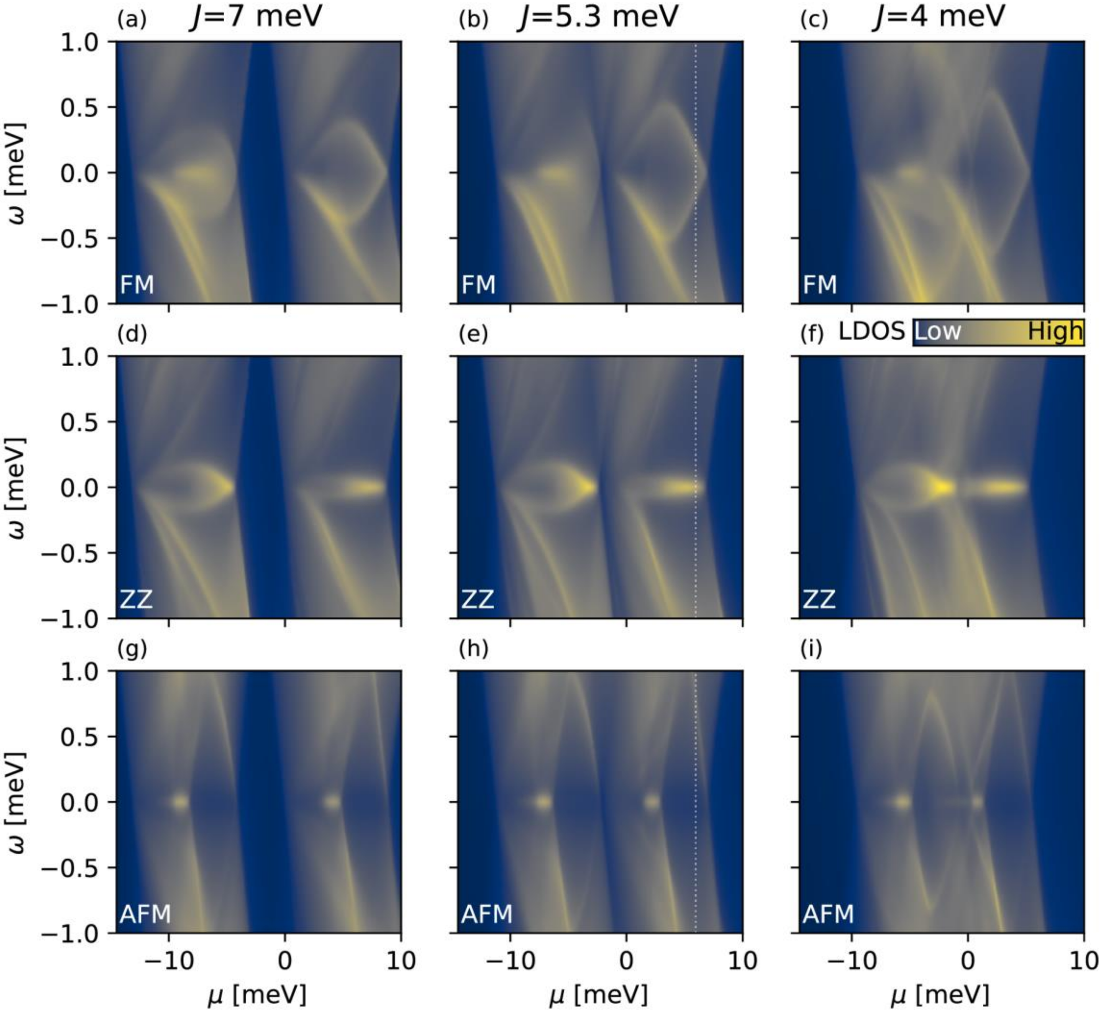

Supplementary Figure 5. Low-energy LDOS for inequivalent types of edges. (a-c) a FM edge, (d-f) a ZZ edge, and (g-h) an AFM edge. The dotted white lines indicate the parameters used in the main text. The parameters used are (a,d,g) J=7 meV, (b,e,h) J=5.3 meV, and (c,f,i) J=4 meV.

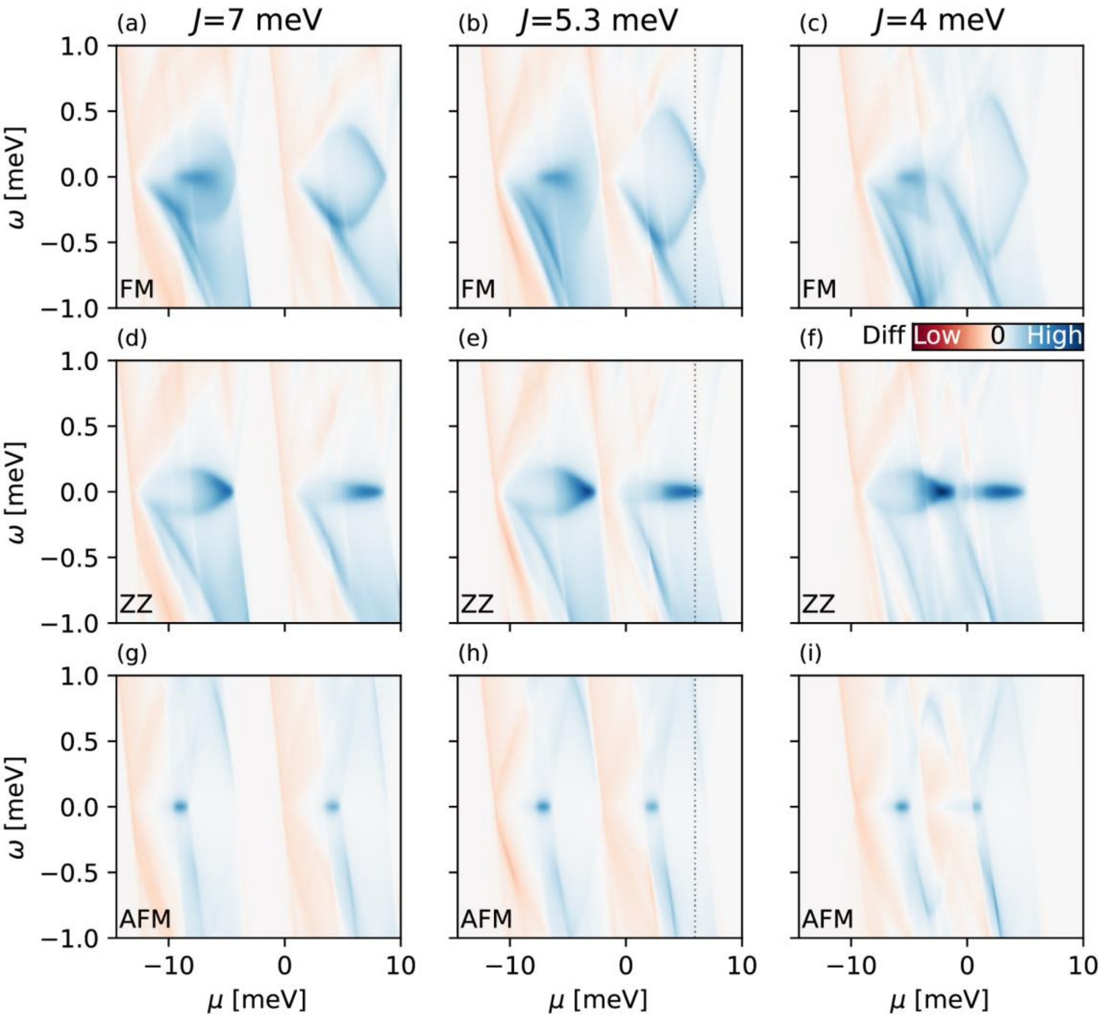

Supplementary Figure 6. Bulk subtracted LDOS for different edges. (a-c) a FM edge, (d-f) a ZZ edge, and (g-i) an AFM edge. The spectral weight remaining after subtracting the bulk LDOS indicates the presence of an edge state. The dotted black lines indicate the parameters used in the main text. The parameters used are (a,d,g) J=7 meV, (b,e,h) J=5.3 meV, and (c,f,i) J=4 meV.

Supplementary Note 4. Modulation of $\alpha^{\pm}$ states along the FM edge

The α state along the FM edge is composed of two very closely positioned peaks, one above and one below the Fermi energy (see Fig. 1e). The spectral feature observed in the experiment resembles a single peak which is actually a superposition of the two components which are not clearly resolved because of our limited energy resolution. Nonetheless, in differential tunneling conductance maps obtained with a superconducting tip, the $\alpha^{\pm}$ states are imaged separately and the antiphase becomes apparent. Supplementary Figure 7 shows a topography of two neighboring islands with three defect free FM edges marked. Differential tunneling conductance maps obtained on each edge and corresponding line profiles extracted from these maps for every edge show the antiphase behavior. The modulation is apparent, however the period is different and it appears to be influenced by other factors from inside of the island or perhaps from the Nb below. The mechanism for this isn't fully understood as the modulation is not captured by the model. For the limited number of edges that we observed, we always see that α^{+} has its maxima at the ends of the edge, and therefore always has one more intensity maximum compared to α^{-}. This could be an indication for a dispersive state, however, a study of many edges in a certain length range would be required to confirm this assumption.

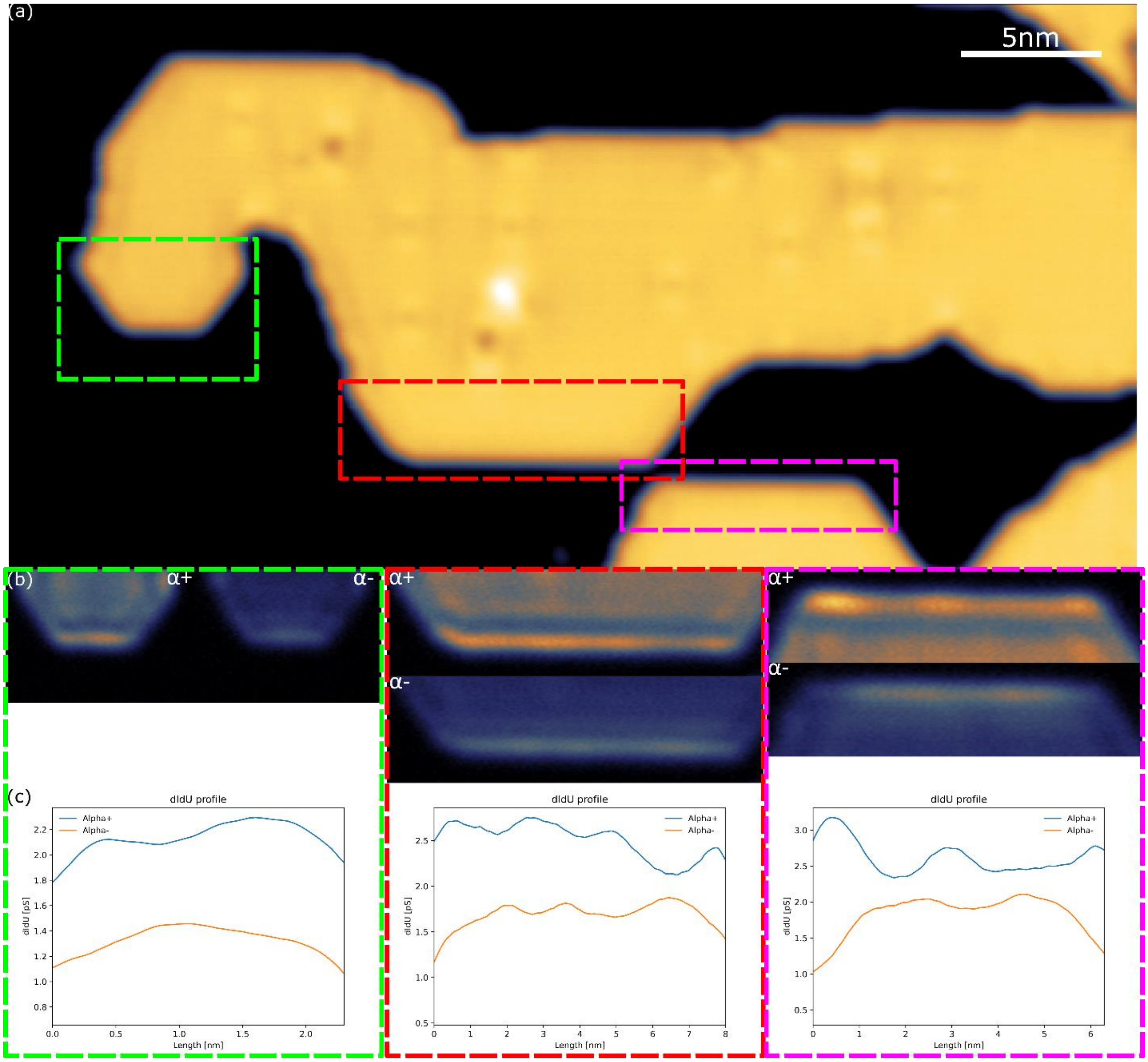

Supplementary Figure 7. Modulation of $\alpha^{\pm}$ states along the FM edge. (a) Topography image of two neighboring Mn islands with defect-free edges marked by rectangles. (b) Differential tunneling conductance maps in marked regions corresponding to α^{+} and α^{-}, respectively. (c) Line profiles extracted from the maps in (b). Tunneling parameters: (a) I_t = 1 nA, U = 50 mV; (b) I_{ts} = 1 nA, U_{stab} = 20 mV, U_{mod} = 100 µV, $Z_{approach}$ = 80 pm, U = ±1.6 mV, Δ_{tip} = 1.41 mV.

Supplementary References

[1] Chiu, C., Teo, J. C. Y., Schnyder, A. P., and Ryu, S. Classification of topological quantum matter with symmetries. *Rev. Mod. Phys.* **88**, 035005 (2016).
